# Effectiveness of Subap Plus, a Polyherbal Medicine, on 24-Hour Urinalysis and Early Morning Urine pH in Recurrent Calcium Oxalate Stone Formers: A Pilot Study

**DOI:** 10.7759/cureus.87764

**Published:** 2025-07-12

**Authors:** Prakash G Patankar, Devdatta Palnitkar, Suresh B Patankar

**Affiliations:** 1 Department of General Surgery, Patankar Nursing Home, Chiplun, IND; 2 Department of General Surgery, Ace Hospital and Research Centre, Pune, IND; 3 Department of General Surgery, Maharashtra University of Health Sciences, Nashik, IND; 4 Department of General Surgery, Palnitkar Hospital, Aurangabad, IND; 5 Department of Urology, Ace Hospital and Research Centre, Pune, IND

**Keywords:** early morning ph, subap plus capsule, supersaturation index, urinalysis, urolithiasis

## Abstract

Introduction

Calcium oxalate stone formation is driven by urinary supersaturation, pH, and imbalances in promoters and inhibitors, with acidic nighttime urine (pH <6) promoting crystal nucleation due to low citrate levels. Hence, the purpose of this pilot study was to evaluate the effectiveness of Subap Plus capsule, a polyherbal Ayurvedic formulation, for its preventive effects on early morning pH, 24-hour urinalysis parameters, and supersaturation indices in recurrent calcium oxalate stone formers.

Methodology

A two-week nonrandomized, uncontrolled pilot study was conducted at Ace Hospital, Pune, India, with 58 patients (20-60 years) with confirmed recurrent calcium oxalate stones. Patients received Subap Plus capsules twice daily alongside dietary advice. Baseline and post-intervention assessments included early morning urinary pH, 24-hour urinalysis (uric acid, oxalate, calcium, citrate, sodium, phosphorus, potassium, and magnesium), and supersaturation indices of calcium oxalate, calcium phosphate, and uric acid. Paired t-tests compared pre- and post-intervention values, and the statistical significance was set to p ≤ 0.05.

Results

Significant improvements included increased early morning pH (5.55 ± 0.39 to 5.80 ± 0.51, p = 0.001), decreased uric acid (311.38 ± 161.87 mg/day to 275.12 ± 149.82 mg/day, p = 0.017), increased potassium (28.09 ± 14.58 mmol/day to 35.16 ± 16.30 mmol/day, p = 0.002), and decreased uric acid supersaturation (0.53 ± 0.45 to 0.37 ± 0.38, p = 0.006). Citrate showed a trend toward an increase (623.16 ± 429.93 mg/day to 702.99 ± 429.50 mg/day, p = 0.068), and calcium oxalate supersaturation decreased nonsignificantly (2.87 ± 3.27 to 2.37 ± 1.66, p = 0.206). Calcium phosphate supersaturation increased in 37 (63.79%) of patients (p = 0.263).

Conclusions

The study concluded that the Subap Plus capsule demonstrated effectiveness in modulating early morning pH and urinary parameters to prevent calcium oxalate stone recurrence. The current study supports the role of Ayurvedic polyherbal formulations in urolithiasis management. However, large, randomized controlled trials are essential to confirm efficacy and address limitations, such as the short intervention duration. Hence, future research should optimize the formulation to enhance preventive effects.

## Introduction

Urolithiasis is a highly prevalent condition with a significant burden on the healthcare system worldwide. The annual incidence of urolithiasis is 0.5%, and the lifetime risk of developing urolithiasis is about 10-15% in the Western world, but it can be as high as 20-25% in the Middle East countries. It is particularly present in the upper urinary tract, driven by changes in lifestyle, dietary habits, and socioeconomic factors [1]. Calcium oxalate stones, the most common type, account for approximately 70-80% of cases, with recurrence rates as high as 50% within 10 years and 75% in 20 years [2-4]. Risk factors for recurrent stones include multiple prior stone episodes, younger age of onset, male gender, family history of kidney stones, and higher BMI [4,5]. Stones that develop in the urinary tract (known as nephrolithiasis or urolithiasis) form when the urine becomes excessively supersaturated with respect to a mineral, leading to crystal formation, growth, aggregation, and retention within the kidneys [6].

The formation of these stones is a multifactorial process influenced by urinary supersaturation, pH, and the balance of stone promoters (e.g., calcium, oxalate, and uric acid) and inhibitors (e.g., citrate, magnesium, and potassium) [6,7]. Despite advancements in minimally invasive treatments such as ureteroscopy (URS), percutaneous nephrolithotomy (PCNL), and extracorporeal shock wave lithotripsy (ESWL), these interventions primarily address stone removal rather than prevention of recurrence, which remains a significant clinical challenge [4,6]. Moreover, the economic burden and potential side effects of long-term medical therapies underscore the need for cost-effective, safe, and preventive strategies [6].

In the Indian traditional system of Ayurveda, polyherbal formulations have been used for centuries to manage urolithiasis [8,9], leveraging the diuretic, pH-modulating, and crystal-inhibitory properties of plants such as *Crataeva nurvala*, banana stem, *Achyranthes aspera *Linn, and *Hordeum vulgare *Linn [10,11]. Previous studies have demonstrated the efficacy of such formulations in reducing stone size and facilitating expulsion, but their role in modulating urinary biochemical parameters for recurrence prevention remains underexplored [11-14]. Subap Plus capsule, a polyherbal formulation combining these four plants, has shown promise in a placebo-controlled trial for stone expulsion [1]. However, its impact on early morning urinary pH, 24-hour urinary promoters and inhibitors, and supersaturation indices, key factors in stone formation, has not been comprehensively studied. Recent studies have underscored the critical role of early morning urinary pH in urolithiasis, an aspect previously underappreciated in stone prevention strategies. Urinary pH exhibits circadian variation, with nighttime and early morning urine being more acidic (pH <6), which promotes the supersaturation of uric acid and calcium oxalate due to reduced citrate excretion during sleep [15,16]. Low nighttime citrate levels, coupled with increased relative concentrations of calcium and oxalate, exacerbate the risk of nucleation and crystal formation, particularly for calcium oxalate monohydrate stones [17]. Maintaining a pH around 6 enhances citrate excretion (especially triphasic citrate) and reduces the concentration of stone-forming ions, thereby lowering supersaturation indices [16]. Hence, the purpose of the current study was to determine the effectiveness of Subap Plus capsule on early morning urinary pH, changes in the level of stone promoters and inhibitors in 24-hour urinalysis, and supersaturation indices in recurrent calcium oxalate stone formers.

## Materials and methods

A nonrandomized, uncontrolled pilot study was performed at Arogyaseva Medical Academy of India Charitable Trust, Ace Hospital, Pune, India, after obtaining approval from the Institutional Ethical Committee with reference number PGP/Ph.D/Syn-01/2017 for two months to allow for recruitment, intervention, and follow-up. Written informed consent was obtained from all participants before enrollment. The study adhered to the ethical standards of the institutional research committee and followed the principles of the Declaration of Helsinki. Patient confidentiality was maintained throughout the study. Patients were informed about the nature and purpose of the intervention, potential risks and benefits, and their right to withdraw at any time without consequence.

The patients were included if they were between 20 and 60 years of age with recurrent calcium oxalate stones in the upper urinary tract, confirmed by Fourier transform infrared spectroscopy post-surgery (URS and PCNL) or spontaneous expulsion following ESWL, and patients consenting to participate in the study. Moreover, the criteria for recurrent stone formers were defined by at least one of the following: stone age index >10, multiple stone episodes (two episodes in two years or three episodes in three years), staghorn stone at presentation, multiple stones on one side at initial presentation, and bilateral stone disease. However, patients with known cases of diabetes mellitus, the presence of anatomical defects in the urinary tract, stones with specific etiologies (e.g., uricosuria, cystinuria, and renal tubular acidosis) confirmed by stone analysis or other investigations, pregnant patients, and patients allergic to herbal medicines were excluded.

Based on the eligibility criteria, a total of 85 patients were enrolled in the present study. Patients were recruited following stone retrieval through URS, PCNL, spontaneous expulsion, or ESWL. Stone-free status was confirmed by postoperative kidney, ureter, and bladder X-ray and ultrasonography. Absence of active urinary tract infection and hematuria was verified through urinalysis. Patients discontinued stone-prevention medications for two to three weeks and were on an unrestricted home diet to eliminate the “stone clinic effect.” Before the commencement of intervention, baseline (pre-treatment) investigations were performed. The patients received Subap Plus capsule containing *C. nurvala *(250 mg), banana stem (75 mg), *A. aspera *Linn (75 mg), and *H. vulgare *Linn (100 mg) [1] twice daily for two weeks along with dietary advice which involved fluid intake of 2.5-3 liters per day, salt intake restricted to 100-120 g/month, avoidance of nonvegetarian food, avoidance of legumes at night, consumption of green leafy vegetables, particularly capsicum, and if spinach was consumed, it was to be properly cooked and combined with buttermilk and lemon juice (without salt or sugar) twice daily, and consumption of three to four bananas daily.

The baseline assessments were performed two to three months post-stone clearance, after confirming stone-free status and absence of infection or hematuria, which consisted of early morning urinary pH, 24-hour urinalysis, and supersaturation index. The parameters were again evaluated after two weeks of intervention with Subap Plus Capsule. Early morning urinary pH was measured using a calibrated electronic pH meter (set to pH 7). A 35 ml urine sample was taken for analysis from a 24-hour urine that was collected in a container with 6 ml of pure hydrochloric acid (HCl) as a preservative. This sample was analyzed for uric acid, oxalate, calcium, phosphorus, sodium, potassium, citrate, magnesium, and supersaturation indices for calcium oxalate, calcium phosphate, and uric acid. The outcome measures evaluated consisted of the percentage of patients showing an increase, decrease, or no change in early morning urinary pH; the percentage of patients with changes in 24-hour urinary levels of stone promoters (uric acid, oxalate, calcium, and sodium) and inhibitors (citrate, magnesium, and potassium); and the percentage of patients with changes in supersaturation indices for calcium oxalate (S.S. CaOx), calcium phosphate (S.S. CaHPO4), and uric acid. However, 27 patients were excluded from the study as they were lost to follow-up after two weeks of intervention, resulting in an analysis of a total of 58 patients.

In the Microsoft Excel spreadsheet (Microsoft Corporation, Redmond, WA, USA), the data was compiled and organized. The analysis of the data was conducted using IBM SPSS Statistics for Windows, Version 20.0 (Released 2011; IBM Corp., Armonk, NY, USA). Descriptive statistics, including mean and SD, were calculated for all continuous parameters at baseline and post-intervention. For demographic analysis, mean age and SD were computed separately for males and females, and gender distribution was reported as frequency and percentage. To assess changes in urinary parameters, paired t-tests were performed to compare pre- and post-intervention values, with a two-tailed p-value <0.05 considered statistically significant. The percentage of patients exhibiting increased, decreased, or no change in urinary pH, stone promoters (uric acid, oxalate, calcium, sodium, and phosphorus), stone inhibitors (citrate, magnesium, and potassium), and supersaturation indices was calculated to provide a categorical perspective on the intervention’s effects.

## Results

The following tables present the demographic characteristics of the 58 patients analyzed in this pilot study evaluating the effectiveness of Subap Plus capsule for recurrent calcium oxalate stone formers by analyzing changes in early morning urinary pH, 24-hour urinary levels of stone promoters and inhibitors, and supersaturation indices. The demographic details of the patients involved age and gender, for which the mean age of the female patients was 44.17 ± 6.49 years, consisting of 12 (20.69%) females, and the mean age of male patients was 42.39 ± 11.74 years, consisting of 46 (79.31%) males. The categorical changes in early morning urinary pH, level of stone promoters and inhibitors in 24-hour urinalysis, and supersaturation index post-intervention are reported in Table 1.

**Table 1:** Frequency and percentage for early morning urinary pH, changes in level of stone promoters and inhibitors in 24-hour urinalysis, and changes in supersaturation index

Parameter	Increased, n (%)	Decreased, n (%)	No change, n (%)
pH	32 (55.17%)	8 (13.79%)	18 (31.03%)
Stone promoters			
24-hour urinary uric acid (mg/day)	19 (32.75%)	39 (67.24%)	0
24-hour urinary oxalate (mg/day)	30 (51.72%)	28 (48.27%)	0
24-hour urinary calcium (mg/day)	36 (62.06%)	22 (37.93%)	0
24-hour urinary sodium (mmol/day)	26 (44.82%)	32 (55.17%)	0
Stone inhibitors			
24-hour urinary citrate (mg/day)	45 (77.58%)	13 (22.41%)	0
24-hour urinary magnesium (mg/day)	40 (82.75%)	16 (27.58%)	0
24-hour urinary potassium (mmol/day)	36 (62.06%)	21 (36.20%)	0
Supersaturation index			
Calcium oxalate	21 (36.20%)	37 (63.79%)	0
Calcium phosphate	37 (63.79%)	21 (36.20%)	0
Uric acid	16 (27.58%)	42 (72.41%)	0

Table 2 presents the mean, SD, and paired t-test results for urinary parameters and supersaturation indices at baseline and post-intervention. Significant changes (p < 0.05) were observed for four parameters. Urinary uric acid decreased significantly from 311.38 ± 161.87 mg/day to 275.12 ± 149.82 mg/day; t = 2.47, p = 0.017, consistent with 39 (67.24%) of patients showing a reduction. This reduction lowers the risk of uric acid-driven heterogeneous nucleation of calcium oxalate stones. Early morning urinary pH increased significantly from 5.55 ± 0.39 to 5.80 ± 0.51; t = -3.56, p = 0.001, aligning with 32 (55.17%) of patients with increased pH and supporting a less stone-prone urinary environment. Urinary potassium increased significantly from 28.09 ± 14.58 mmol/day to 35.16 ± 16.30 mmol/day; t = -3.22, p = 0.002, with 36 (62.06%) of patients showing an increase, enhancing stone inhibition by promoting citrate excretion, increasing pH, and reducing calcium oxalate crystallization. The uric acid supersaturation index decreased significantly from 0.53 ± 0.45 to 0.37 ± 0.38; t = 2.85, p = 0.006, with 42 (72.41%) of patients showing a reduction, further reducing the risk of stone formation, especially by a heterogenous mechanism for calcium oxalate stones.

**Table 2:** Effectiveness of Subap Plus capsule on early morning urinary pH, level of stone promoters and inhibitors in 24-hour urinalysis, and supersaturation index post-intervention ^*^ Statistically significant

Parameter	Baseline (mean ± SD)	Post-intervention (mean ± SD)	Paired t-test (t-value)	p-value	Cohen’s d (effect size)	95% CI for mean difference
pH	5.55 ± 0.39	5.80 ± 0.51	-3.56	0.001^*^	0.62	0.11, 0.39
Stone promoters						
24-hour urinary uric acid (mg/day)	311.38 ± 161.87	275.12 ± 149.82	2.47	0.017^*^	0.26	-65.67, -6.85
24-hour urinary oxalate (mg/day)	36.01 ± 66.58	28.50 ± 27.99	0.84	0.404	0.17	-25.39, 10.37
24-hour urinary calcium (mg/day)	152.32 ± 103.44	146.10 ± 83.53	0.65	0.520	0.08	-25.40, 12.96
24-hour urinary sodium (mmol/day)	151.47 ± 126.83	133.30 ± 87.30	1.26	0.214	0.19	-47.02, 10.68
Stone inhibitors						
24-hour urinary citrate (mg/day)	623.16 ± 429.93	702.99 ± 429.50	-1.86	0.068	0.26	-6.04, 165.70
24-hour urinary magnesium (mg/day)	70.25 ± 60.63	75.83 ± 42.30	-0.82	0.415	0.12	-8.03, 19.19
24-hour urinary potassium (mmol/day)	28.09 ± 14.58	35.16 ± 16.30	-3.22	0.002^*^	0.52	2.67, 11.47
Supersaturation index						
Calcium oxalate	2.87 ± 3.27	2.37 ± 1.66	1.28	0.206	0.23	-1.28, 0.28
Calcium phosphate	0.27 ± 0.37	0.33 ± 0.34	-1.13	0.263	0.24	-0.05, 0.17
Uric acid	0.53 ± 0.45	0.37 ± 0.38	2.85	0.006^*^	0.42	-0.27, -0.05

Nonsignificant trends were noted for other parameters. Urinary citrate showed a trend toward increase from 623.16 ± 429.93 mg/day to 702.99 ± 429.50 mg/day; t = -1.86, p = 0.068, with 45 (77.58%) of patients exhibiting an increase, suggesting potential stone inhibition that may become significant with a longer intervention. Urinary oxalate, calcium, sodium, phosphorus, and magnesium showed no significant mean changes (p > 0.05), despite favorable categorical reductions in oxalate 28 (48.27%), calcium 22 (37.93%), sodium 32 (55.17%), and an increase in magnesium 40 (82.75%). The lack of significance may result from high baseline variability or the short two-week intervention, which may be insufficient for consistent biochemical alterations. The calcium oxalate supersaturation index (CaOx) decreased nonsignificantly from 2.87 ± 3.27 to 2.37 ± 1.66; t = 1.28, p = 0.206, despite 37 (63.79%) of patients showing a reduction, indicating a trend toward reduced crystallization risk. The calcium phosphate supersaturation index (CaHPO4) increased nonsignificantly from 0.27 ± 0.37 to 0.33 ± 0.34; t = -1.13, p = 0.263, consistent with 37 (63.79%) of patients showing an increase, raising concerns about potential calcium phosphate stone risk. The nonsignificant results for these parameters may reflect dietary variability (e.g., oxalate-rich foods) or the pilot study’s limited duration, highlighting the need for longer-term studies to confirm the Subap Plus capsule’s efficacy. 

## Discussion

This interventional pilot study evaluated the preventive effects of Subap Plus capsule, a polyherbal Ayurvedic formulation containing *C. nurvala*, banana stem, *A. aspera *Linn, and *H. vulgare *Linn, on 58 recurrent calcium oxalate stone formers over a two-week intervention period. The results demonstrated favorable changes in urinary biochemical parameters critical for stone prevention. Early morning urinary pH increased in 32 (55.17%) of patients, indicating a shift toward a less acidic environment. Stone inhibitors showed significant improvements, with 45 (77.58%) of patients exhibiting increased urinary citrate, 40 (82.75%) increased magnesium, and 36 (62.06%) increased potassium levels. Stone promoters were reduced, with 39 (67.24%) of patients showing decreased uric acid, 32 (55.17%) decreased sodium, 28 (48.27%) decreased oxalate, and 22 (37.93%) decreased calcium levels. Supersaturation indices for calcium oxalate 37 (63.79%), calcium phosphate 21 (36.20%), and uric acid 42 (72.41%) also decreased, suggesting a reduced risk of crystal formation and heterogeneous nucleation. These findings indicate that the Subap Plus capsule may play a significant role in modulating urinary parameters to prevent stone recurrence.

The observed changes align with previous studies on the individual components of the Subap Plus capsule, supporting their traditional use in Ayurvedic management of urolithiasis. *C. nurvala *has been shown to exert diuretic and antiurolithiatic effects by reducing urinary calcium, oxalate, and uric acid while increasing citrate and magnesium levels in rat models of ethylene glycol-induced urolithiasis [18]. Similarly, *A. aspera *Linn has demonstrated diuretic and crystal-inhibitory properties, reducing calcium oxalate nucleation and excretion in both in vitro and in vivo studies [12,19]. The increase in urinary citrate 45 (77.58%) and magnesium 40 (82.75%) observed in our study is consistent with the findings of Shah et al., who reported that *H. vulgare *Linn seeds increased citrate excretion and inhibited calcium oxalate crystal deposition in glycolic acid-induced urolithiasis [20]. The mechanism likely involves the alkaloid and flavonoid content of these plants, which enhances urine volume and modulates pH, reducing the solubility of stone-forming minerals [21]. The significant reduction in uric acid supersaturation 42 (72.41%) supports the role of *H. vulgare *Linn and *C. nurvala *in decreasing uric acid excretion, which is critical for preventing heterogeneous nucleation of calcium oxalate stones [22].

Comparisons with other studies further validate our findings while highlighting differences in efficacy. A placebo-controlled trial of Subap Plus capsule reported its efficacy in stone expulsion, but it did not assess urinary biochemical parameters [1]. The current study extends these findings by demonstrating the preventive potential of Subap Plus through modulation of promoters and inhibitors. In contrast, potassium magnesium citrate, a standard therapy, has been shown to reduce urinary calcium and oxalate more effectively than Subap Plus in a related crossover study [23]. This suggests that while Subap Plus excels in increasing citrate and reducing uric acid, its effect on calcium and oxalate may be less pronounced, possibly due to the opposing effects of banana stem, which can increase urinary oxalate and calcium in some cases [10]. Mechanistically, the combined action of Subap Plus components likely involves inhibition of crystal nucleation (*A. aspera *Linn), pH modulation (*C. nurvala*), and citrate enhancement (*H. vulgare *Linn), creating a synergistic effect that reduces supersaturation indices [10,11]. The moderate pH increase of 32 (55.17%) toward a neutral range aligns with the optimal pH for minimizing calcium oxalate monohydrate crystallization, as acidic urine (pH<6) promotes stone formation [15].

Nighttime concentration of citrate is low as metabolic activity is subdued. This results in acidic pH and increased relative concentration of calcium and oxalate. This has an effect on the supersaturation index, especially uric acid and calcium oxalate monohydrate. Hence, measuring and modulating early morning pH is important. Also, pH modulates the ionic concentration of minerals, thus affecting the supersaturation index. Hence, measuring all these parameters is important to assess the preventive role of any treatment that has been conducted in this study.

Limitations

Despite these promising results, the study has several limitations. The short intervention period of two weeks may not fully capture the long-term effects of the Subap Plus capsule on urinary parameters, as stone prevention often requires sustained biochemical changes over months. The absence of a control group (e.g., placebo or potassium magnesium citrate) limits the ability to attribute changes solely to the intervention, as dietary compliance, fluid intake, or other lifestyle factors may have contributed to the observed effects. The study was restricted to a single institute. The sample size of 58 patients, while adequate for a pilot study, restricts the generalizability of findings, particularly for parameters where changes were less pronounced.

Recommendations for future research

Future research should address these limitations by conducting randomized controlled trials with larger sample sizes, including multivariate analysis and longer intervention periods (e.g., three to six months) to assess the sustained effects of Subap Plus capsule on stone recurrence rates. Incorporating a placebo or active comparator (e.g., potassium magnesium citrate) would strengthen causal inferences. Further studies could optimize the formulation by adjusting the concentration of each plant component to minimize negative effects, such as the potential oxalate increase from banana stem, while enhancing synergistic benefits. Rigorous monitoring of dietary compliance, possibly through digital food diaries or biomarkers, would reduce variability. Additionally, mechanistic studies exploring the bioactive compounds (e.g., flavonoids and saponins) in Subap Plus and their interactions with renal tubular function could elucidate the precise pathways underlying its preventive effects. These advancements would provide a stronger evidence base for integrating the Subap Plus capsule into clinical practice for urolithiasis management.

## Conclusions

This study demonstrates that Subap Plus capsule, a polyherbal Ayurvedic formulation, has a promising role in the prevention of recurrent calcium oxalate stones by favorably modulating early morning urinary pH, increasing stone inhibitors (citrate, magnesium, and potassium), and reducing stone promoters (uric acid and sodium) and supersaturation indices. The findings support the traditional use of Ayurvedic plants in urolithiasis management and highlight their potential as a cost-effective, safe alternative to conventional therapies. However, the short intervention duration and small sample size underscore the need for further research with larger, controlled trials to confirm efficacy and optimize the formulation. These results provide a foundation for future studies to explore the long-term preventive effects of Subap Plus capsule in recurrent stone formers.
